# Assessment of risk factors in the development of MRSA infection at Gülhane Military Medical Faculty Education Hospital and the role of antibiotic use on the development of MRSA infection

**DOI:** 10.1186/1753-6561-5-S6-P14

**Published:** 2011-06-29

**Authors:** E Gunal, BA Besirbellioglu, F Ersoz, IY Avci, CP Eyigun

**Affiliations:** 1 Infectious Disease and Clinical Microbiology, Gulhane Military Medical Academy, Turkey; 2 Defense Science Institue, Turkish Military Academy, Ankara, Turkey

## Introduction / objectives

This research was carried out to determine most important risk factors in the development of MRSA by comparing infections due to methicillin resistant and susceptible strains to make suggestions to decrease MRSA ratios by pointing out the importance of pre-infection antibiotic usage in the development of MRSA infection and its place among the other risk factors.

## Methods

This research was planned as a retrospective case control study and possible risk factors among inpatients owing to *S. aureus* infection at our hospital between 2003-2008 years, were compared as MRSA and MSSA groups.

## Results

Hospitalization period, previous hospitalization existence and number, lining in intensive care unit, MRSA existence in the same unit, polymicrobial infection, acute trauma, surgery, open lesion, any intravenous, urethral catheter, mechanical ventilation, invasive device number, previous antibiotic usage, number and period of used antibiotic were found more significant in MRSA patients(p<0,05).Respectively, flouroquinolone usage (OR, 2,56; %95 CI: 1,052–6,231; p<0,05), the time period of previous antibiotic use (OR, 2,343; %95 CI: 1,697–3,236; p<0,05), hospitalization times (OR, 1,396; %95 CI: 1,235–1,578; p<0,05), previous hospitalization period (OR, 0,992; %95 CI: 0,986–0,999; p<0,05), MRSA existence in the same milieu (OR, 0,283; %95 CI: 0,13–0,618; p<0,05) were determined as independent risk factors in the development of infections due to MRSA.

## Conclusion

Controlling these risk factors and either avoiding uncontrolled prescription or decreasing the use of selected antibiotic subcategories like flouroquinolones and cephalosporins seem to reduce infection due to MRSA.

## Disclosure of interest

None declared.

